# Home-based training program in patients with chronic heart failure and reduced ejection fraction: a randomized pilot study

**DOI:** 10.6061/clinics/2021/e2550

**Published:** 2021-05-27

**Authors:** Geisa Nascimento de Andrade, Iracema Ioco Kikuchi Umeda, Angela Rubia Cavalcanti Neves Fuchs, Luiz Eduardo Mastrocola, João Manoel Rossi-Neto, Dalmo Antonio Ribeiro Moreira, Patricia Alves de Oliveira, Carmen Diva Saldiva de André, Lawrence Patrick Cahalin, Naomi Kondo Nakagawa

**Affiliations:** I Departamento Fisioterapia, Faculdade de Medicina FMUSP, Universidade de Sao Paulo, Sao Paulo, SP, BR.; II Instituto Dante Pazzanese de Cardiologia, Sao Paulo, SP, BR.; III Divisao de Cardiologia, Instituto do Coracao (InCor), Hospital das Clinicas HCFMUSP, Faculdade de Medicina, Universidade de Sao Paulo, Sao Paulo, SP, BR.; IV Departamento de Estatistica, Instituto de Matematica e Estatistica, Universidade de Sao Paulo, Sao Paulo, SP, BR.; V Department of Physical Therapy, University of Miami, Miller School of Medicine, Florida, USA.

**Keywords:** Cardiac Rehabilitation, Telerehabilitation, Heart Failure, Endurance Training, Resistance Training, Exercise

## Abstract

**OBJECTIVES:**

We aimed to compare the effects of home-and center-based exercise training programs on functional capacity, inspiratory muscle strength, daily physical activity level, and quality of life (QoL) in patients with chronic heart failure (CHF) over a 12-week period.

**METHODS:**

This study included 23 patients with CHF (left ventricular ejection fraction 31±6%) randomized to a home-based (n=11) or center-based (n=12) program. Patients underwent 12 weeks of aerobic training (60%-70% heart rate reserve): walking for the home-based and supervised cycling for the center-based group, both combined with resistance training (50% of 1 maximum repetition). At baseline and after 12 weeks of training, we assessed cardiopulmonary test variables, 6-min walk test distance (6 MWD), steps/day with accelerometry, and QoL (Minnesota Living with Heart Failure questionnaire). Maximal inspiratory pressure and handgrip strength were measured at baseline and after 4, 8, and 12 weeks of training. ClinicalTrials.gov: NCT03615157.

**RESULTS:**

There were no adverse events during training in either group. The home- and center-based training groups obtained similar improvements in peak oxygen uptake, maximal ventilation, and 6 MWD. However, there were significant between-group differences: center-based training was more effective in improving maximal inspiratory pressure (*p*=0.042), number of steps/day (*p*=0.001), and QoL (*p*=0.039).

**CONCLUSIONS:**

Home-based training is safe and can be an alternative to improve the exercise capacity of patients with stable CHF. However, center-based training was superior in improving inspiratory muscle strength, QoL, and daily physical activity.

## INTRODUCTION

Chronic heart failure (CHF), the end stage of several cardiac diseases, is increasing worldwide (1). Patients with this syndrome display symptoms such as dyspnea, fatigue, and exercise intolerance that limit their activities in daily life (2). Physical exercise improves functional capacity, quality of life (QoL), and inspiratory muscle strength and reduces cardiac events and hospitalizations in patients with CHF (3,4). However, center-based exercise adherence depends on factors such as time expenditure, accessibility limitations, financial costs, work and/or domestic commitments, and restricted availability of cardiac rehabilitation centers (5), particularly in low-and middle-income countries (6). With the advent of the coronavirus 2019 pandemic, when social distancing is necessary to prevent transmission, traditional center-based cardiac rehabilitation has become more limited (7,8).

Home-based training can be an alternative to supervised training for patients with stable CHF (9). Several home-based programs have been reported in the literature. Some of these comprised education, phone call support, and instructions that can be combined with resistance exercises (10), aerobic exercises, walking (11-13), or using a cycle ergometer or treadmill (14). Other home-based programs have combined aerobic and resistance exercises (15), and some have added telemonitoring to walking (16) or resistance exercises (17). Compared with non-training, home-based programs have shown benefits for exercise capacity (12,14) and QoL (12-15). Compared with traditional center-based training, home-based programs with telemonitoring have demonstrated similar results for exercise capacity, QoL, and exercise safety in patients with CHF (16,17).

In this respect, the use of home-based training programs as an easy, simple, and low-cost alternative to supervised center-based training for patients with stable CHF may be useful, especially in poor and middle-income countries. In the present study, we hypothesized that a simple and easy-to-perform unsupervised home-based training program may have similar effects to its traditional supervised center-based counterpart. We aimed to compare the effects of a home-based with a center-based training program on exercise capacity, physical activity level, QoL, and treatment adherence in patients with CHF.

## MATERIALS AND METHODS

### Trial design

This randomized, controlled, open-label, pilot trial was approved by the University of Sao Paulo Medical School Ethics Committee (reference 411/14) and Dante Pazzanese Institute of Cardiology Ethics Committee (reference 4536), which followed the ethical guidelines of the Declaration of Helsinki. Patients were randomized using sealed envelopes in a 1:1 allocation after written consent was obtained. ClinicalTrials.gov: NCT03615157.

### Participants

Patients aged >18 years with CHF, New York Heart Association (NYHA) functional class II or III (18), and left ventricular ejection fraction of <40% were recruited consecutively from a list of new patients at the HF outpatient of the Dante Pazzanese Institute of Cardiology, from April 2015 to April 2018. The exclusion criteria were patients with new-onset atrial fibrillation or atrial flutter, complex ventricular arrhythmia at rest or presenting with exertion (19), acute or decompensated HF, pulmonary hypertension (pulmonary artery systolic pressure >35 mmHg), any orthopedic, cognitive, or neurological problems that could affect functional capacity measures, respiratory infection in the previous 30 days, and peripheral oxygenation of <92% in ambient air at rest.

### Interventions

**Figure 1:**
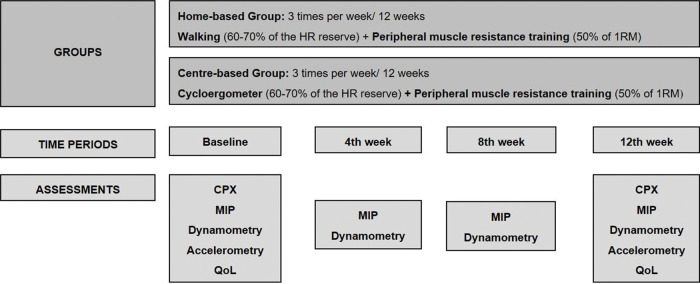
Study design of interventions and assessments. CPX, cardiopulmonary test; HR, heart rate; 1RM, one repetition maximum; MIP, maximal inspiratory pressure; QoL, quality of life.

Following the baseline cardiopulmonary exercise test (CPX), patients were randomized to a home- or center-based training group (Figure 1). Considering the aerobic training, both groups used the same target heart rate (HR) of 60%-70% of the HR reserve (difference between maximum HR at CPX and resting HR) and patients were instructed to maintain their perceived exertion between 10 and 14 of the Borg scale (19). Each subject in both groups used a HR monitor (Polar FT1TM, Polar Electro Oy, Kempele, Finland) over the 12-week training period to ensure the target intensity of aerobic exercise and to avoid work overload. Additionally, both groups also performed resistance exercises at 50% of one repetition maximum (1RM), which was assessed and revised once a month. The 1RM test was performed according to the guidelines of the American College of Sports Medicine (20).

Home-based training comprised walking (three times a week for 30 min) in which patients were instructed to maintain the target HR, combined with resistance exercises guided by an illustrated instruction manual for the upper limbs (elbow flexion and extension, and shoulder flexion and abduction) and lower limbs (hip flexion, extension and abduction, knee extension, and plantar flexion) using free weights. The exercise intensity to initiate the program was one set of ten repetitions that followed a final progression to three sets of ten repetitions for each exercise with 50% of 1RM adjusted monthly over the training period. Free weights were provided for each patient according to the assessments. The patients were trained at least once per month with physiotherapist supervision, and the adherence and HR reached during the walks were monitored on a diary filled by the patients. Furthermore, the researcher made weekly phone calls to stimulate patients to continue performing daily exercises, to screen exercise adherence, and to answer possible doubts.

Center-based training took place at a cardiac rehabilitation facility of a cardiac hospital. The training program was supervised by physiotherapists and comprised cycle ergometer exercises (three times a week for 30 min) to maintain the target HR, and resistance exercises for the upper and lower limbs. A physiotherapist recorded the patientś adherence to each session.

### Outcome measures

Anthropometric data, NYHA functional class, HF etiology, cardiac rhythm (24-h Holter, CardioLight, Cardios^®^, São Paulo, Brazil), presence of comorbidities, smoking status, and the use of medications were collected at baseline.

### Assessments of exercise capacity

Exercise capacity was assessed using the CPX measures and the 6-min walk (6 MW) test at baseline and after the 12-week training period. Patients were instructed to have a light breakfast 2h before exercise capacity assessments, to take their medication, and to abstain from consuming caffeine-containing beverages, tea, and alcohol. The tests were performed between 8 a.m. and 11 a.m.

CPX was performed in a controlled environment (23°C room temperature and 55%-60% relative humidity) by blinded physicians and analyzed according to the European Society of Cardiology Guidelines (21). For this study, CPX was performed on a treadmill using a modified Balke ramp protocol at 2.0-3.4 mph (Trackmaster^®^, Full Vision Inc, KS, USA). Oxygen consumption (VO_2_), carbon dioxide output (VCO_2_), and minute ventilation (VE) were measured continuously breath-by-breath, with the aid of a gas exchange analysis system (Ultima™ CardiO2^®^, MGC Diagnostics Corp, MN, USA). A 12-lead electrocardiogram continuously monitored HR and cardiac rhythm throughout the test.

The 6 MW test was performed on a different day than the CPX. Patients were instructed to walk as fast as possible for 6 min in a 30-m corridor. We used verbal stimulation with standardized phrases every minute, as recommended by the American Thoracic Society guidelines (22). To avoid the learning effect, two tests were performed on the same day, with a minimum interval of 30 min. The test with the longest distance was used for the statistical analysis.

### Assessments of respiratory and peripheral muscle strength

Respiratory and peripheral muscle strength were monitored at baseline and after 4, 8, and 12 weeks of training. The maximum value of three consecutive repetitions of each test was recorded. The inspiratory muscle strength assessment followed American Thoracic Society guidelines (23). Maximal inspiratory pressure was assessed using an analog pressure manometer with a range of ±120 cmH_2_O (WIKA DO BRASIL, Sao Paulo, Brazil). Patients sat in a chair with back support and inhaled forcefully and as quickly as possible in a mouthpiece with a 2 mm leak to avoid glottal closure. The maximal inspiratory pressure was measured at the residual volume. The results are presented as absolute values expressed in cmH_2_O. Inspiratory muscle weakness was defined as a predicted (24) maximal inspiratory pressure of <70% (25).

Peripheral muscle strength was assessed using dominant handgrip strength measured by an analog dynamometer (Jamar Hydraulic Hand Dynamometer, Sammons Preston Inc., Illinois, USA). The procedure followed the recommendations of the American Society of Hand Therapists (26).

### Assessments of QoL, physical activity, and sedentary behavior

Self-reported cardiac disease-specific QoL was assessed at baseline and after 12 weeks of training using the Minnesota Living with Heart Failure Questionnaire (MLHF) (27), in which higher scores represent worse QoL. We also used a validated version of the Short-Form 36 Questionnaire (SF-36) (28), which has eight sub-dimensions analysis ranging from 0 to 100, with lower scores representing worse health-related QoL levels.

For self-reported physical activity level, we used the International Physical Activity Questionnaire (IPAQ)-long form (29), which classifies subjects as sedentary, irregularly active, active, and very active.

We also performed objective measurements of physical activity and sedentary behavior using accelerometers at baseline and after the 12-week training period. Patients wore the device (GT3X Triaxial accelerometer, ActiGraph, FL, US) over their dominant hip for 24h over 9 consecutive days, except when bathing or swimming. For the data to be valid, patients had to wear the device for at least 10 h/day for 3 days. Data were recorded at a frequency of 30 Hz and sample intervals of 60-s epochs. Using the software (Actilife5, ActiGraph, FL, USA), we validated the wear time, converted units (counts) into steps, and classification of physical activity intensities using the Freedson energy expenditure algorithm (30). Sedentary behavior was assessed using sedentary bouts (no activity for more than 10 consecutive minutes) and total sedentary behavior per day. We also assessed the number of steps per day and time spent per day engaging in light and moderate-vigorous activity. Additionally, patients completed a daily report to ensure the wear time of the accelerometer.

### Statistical analyses

This was a pilot study with an expected small sample size. In the present study, we aimed to examine the feasibility of two exercise training programs for use in a larger-scale study. We did not estimate the effect size because of the inherent data imprecision from small samples (31).

We used Minitab^®^ Statistical Software (version 17; State College, PA, USA) and R package (version 3.4.3; Vienna, Austria) for statistical analyses. At baseline, intergroup comparisons of anthropometric data, NYHA functional class, HF etiology, cardiac rhythm, presence of comorbidities, smoking status, and the use of medications were conducted using the Mann-Whitney test or the chi-squared test. To evaluate the effects of time and group×time interactions between the two randomized arms, we used non-parametric analysis and mixed linear models (32). When a group×time interaction was detected, Tukey’s multiple comparison test was applied. The difference in means and 95% confidence intervals were calculated and showed when the effects of the time or group×time interaction were identified. A non-parametric two-way analysis of variance for repeated measures (33) was performed to compare the ordinal data of the IPAQ. A significance level of 0.05 was set for the hypothesis tests.

## RESULTS

**Figure 2:**
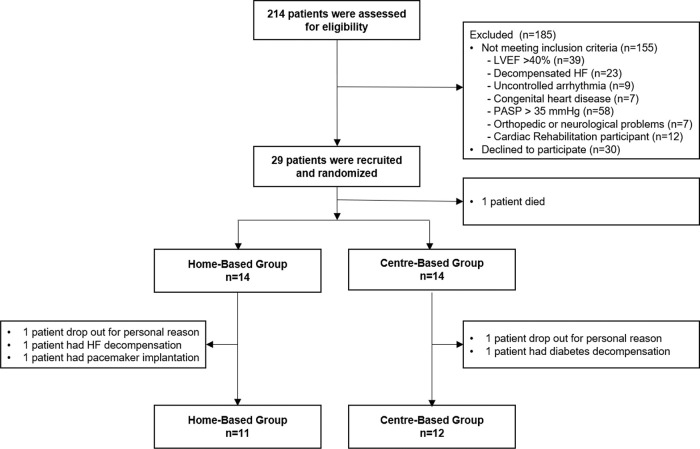
Recruitment and intervention flowchart. HF, heart failure; LVEF, left ventricular ejection fraction; PASP, pulmonary artery systolic pressure.

Twenty-nine patients were enrolled in the study; however, one patient died from a cardiovascular disorder before initiating the training program. Of the 28 patients, only 23 completed the protocol (Figure 2). There were no cardiorespiratory arrests or deaths during either exercise training program.

Patient characteristics such as anthropometric data, NYHA functional class, HF etiology, cardiac rhythm, comorbidities, smoking status, and medication use were similar between the home-based and center-based groups at baseline (Table 1).

Exercise capacity assessed by the CPX was similar in both groups at baseline. All patients reached the target HR during both training programs, maintaining the workload between the aerobic and anaerobic thresholds. Home- and center-based training improved peak VO_2_ (4% and 19%) and maximal ventilation (12% and 16%, respectively), without differences between the groups (Table 2).

**Figure 3:**
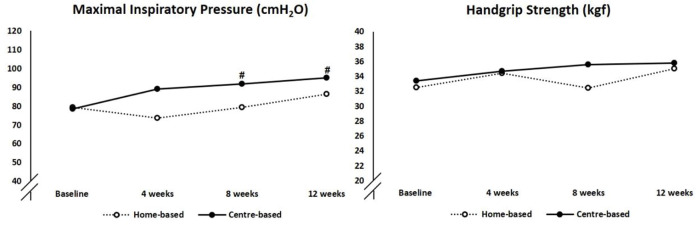
Respiratory and peripheral muscle strength at baseline and at 4, 8, 12 weeks of training. ^#^*vs* baseline, *p*=0.019 using Tukey’s multiple comparison test.

At baseline, two home-based and four center-based patients exhibited inspiratory muscle weakness, and after training, only one patient in the center-based group had inspiratory muscle weakness. The center-based training group showed greater improvement in inspiratory muscle strength than their home-based counterpart (20% and 10%, respectively, *p*-value to between group difference=0.042; Figure 3). There were no changes in handgrip strength in either group.

At baseline, both groups were mostly sedentary and irregularly active. After training, the patients became more active according to the IPAQ analysis (Table 2). Using accelerometers, we observed a significant increase in the number of steps per day in the center-based compared with the home-based group (15% and -7%, respectively, *p*-value to between group difference=0.031). Additionally, the sedentary bout duration of the center- and home-based training groups declined by 14% and 9%, respectively, albeit with no changes in the number of sedentary bouts per day. The time spent per day in the different intensity categories of physical activity remained unchanged after both training programs (Table 2).

Health-related QoL improvement assessed by the MLHF was superior in the center-based group compared with the home-based group. Considering the SF-36 questionnaire results, the mental health component alone had a greater improvement in the center-based group than in the home-based group (Table 2).

The adherence rate in the home-based training program was observed by the daily report of the target HR registration in the diary and weekly phone calls, which showed 89% for walking and 94% for resistance exercises. In the center-based training program, the adherence rate was 94% for both groups. Adherence was similar in both groups (*p*=0.167).

## DISCUSSION

In the present study, we compared an unsupervised home-based program (walking and resistance exercises) with a traditional center-based program (cycling and resistance exercises) in patients with CHF over a 12-week period. Peak VO_2_ and 6 MW distance improved in both groups. However, the supervised center-based program was more effective in improving daily physical activity, inspiratory muscle strength, and QoL in patients with CHF. Furthermore, no adverse events were observed in either group.

Exercise plays a crucial role in cardiac rehabilitation. However, several factors can affect patient participation and adherence to center-based programs (5,6,8). Home-based training has emerged as a safe, simple, easy-to-perform, and low-cost alternative to center-based training, as reported by other studies using walking (11,13) and resistance exercises (10,17). Studies comparing home-based and traditional center-based programs have shown similar benefits for exercise capacity (13,16,17). Our study showed that both training programs had high adherence and improved exercise capacity over 12 weeks. Each program had barriers and facilitators to promote patient adherence. Our center-based program was performed at a public specialized cardiac center that required patients’ fidelity to the program (a maximum of two absences/month were allowed). Additionally, multidisciplinary work teams and group activities may increase patients’ confidence, motivation, and adherence. However, the costs and time for displacement, work release for center-based training, and personal dislike of group activities may limit adherence. On the other hand, the home-based program was freely adapted to the patients’ schedules, and there were no additional transport costs and no need for work release. Nevertheless, home-based programs require patients’ self-motivation, self-discipline, and schedule organization.

Although there was no statistical difference between the groups, the peak VO_2_ improved by 19% in the center-based group and 4% in the home-based group. It is possible that participants did not keep up with the target HR during the 30 min of walking, despite the patients’ HR and adherence being monitored by the researchers with weekly phone calls and a diary filled by them after daily exercises. Another possibility is that participants in the center-based group trained about 10% above the target HR using cycle ergometers, as CPX was performed on treadmills. However, all the patients were safely trained below 80% of the maximum HR. Additionally, it should be noted that our results are similar to those of other studies. Piotrowicz et al. (16) performed CPX in treadmills for prescription of aerobic exercise using cycle ergometers for supervised training and walking for home-based training. They found similar improvements in peak VO_2_ in the supervised training (6%) and home-based groups (10%). Our protocol for both training programs included peripheral resistance training that was not prescribed in the study by Piotrowicz et al. (16). We raise the possibility that resistance training may also have contributed to this improvement in peak VO_2_ in the center-based group (19%).

On the other hand, the submaximal exercise capacity, assessed by the 6 MW distance improved in both groups (the home-based and center-based groups improved by 40 m and 25 m, respectively). Only the home-based group reached the minimal important difference in the 6 MW distance of 36 m, which was previously reported in stable systolic CHF patients (34). The fact that walking was the modality of aerobic training in the home-based group may have favored the performance of the patients in the 6 MW test.

Respiratory and peripheral muscle weakness are abnormalities that lead to deleterious disuse atrophy and physical inactivity in CHF (25,35). Aerobic exercise may partially reverse weakness or improve the respiratory muscle strength (4,36). In our study, the improvement in inspiratory muscle strength in the center-based group was significantly superior to that in the home-based group (20% and 10%, respectively). Considering that the target HR was set based on the CPX performed on the treadmill, the center-based group may have been trained with a load 10% above the home-based group, as the training was on cycle ergometers. This intensity difference may have influenced the superior improvement in inspiratory muscle strength in the center-based group.

Handgrip strength in patients with CHF is a predictor of mortality when values are lower than 32.2 kgf (37). In the present study, at baseline, we observed that approximately 50% of patients in both groups had a handgrip strength less than this predictor value. After the 12-week training period, only 10%-15% of patients improved their upper muscle strength enough to exceed this clinical cutoff value**.** We raise the possibility that despite prescribing the recommended load for the strength exercises (50% of 1RM) (19) that was adjusted monthly for each patient, the training intensity and volume performed may not have promoted sufficient improvement in muscle strength. Additionally, aerobic exercises with the use of a cycle ergometer or walking did not directly contribute to the muscle strength of the upper limbs.

Both groups exhibited different walking behaviors (number of steps/day) at baseline and after training. At baseline, the center-based and home-based patients walked 5,640 and 7,345 steps/day, respectively. After training, the number of steps/day increased significantly in the center-based group (15%), although the home-based group showed no change. This improvement in the number of steps/day in the center-based group is associated with a change in the habit of these patients. As the training modality was the cycle ergometer, the center-based patients became more active in their daily living and not only in the exercise training sessions. Additionally, participants in the center-based training group had to move from home to the center three times per week, which could have affected the marked increase in their number of steps compared with participants in the home-based group.

Considering the self-report, most of our patients changed their perception of physical activity performance. At baseline, they were classified as sedentary or irregularly active by the IPAQ, and after the training programs, almost 100% of patients from both groups self-reported being active, which was corroborated by objective measurements showing reduction in sedentary behavior. This improvement in self-reported physical activity may be related to the amelioration in exercise capacity observed in both groups and consequently an increase in the patients’ self-confidence in engaging in physical activity.

We found that the center-based group had a significant improvement in the QoL, observed by a 13-point decrease in the MLHF and an increase in the mental health component of the SF-36 questionnaire. The social involvement and multidisciplinary approach during the supervised exercises in the group may have contributed to this QoL improvement. Other studies did not observe differences between center-based and home-based training programs (10,17).

Our study had some limitations. This was a pilot, single-center study, with a final small sample size that may have been underpowered for accurate comparisons of functional capacity between the two programs. However, we used accurate and well-validated measures for the domains of interest. Despite the small number of patients in each group, we found both programs safe, as we did not have any reports or objective measurements of adverse events such as tachycardia, arrhythmia, or any signals or symptoms of low cardiac output. The home-based program might not reach the same efficiency in cardiovascular training as the center-based program, which showed greater improvements in maximal exercise capacity, inspiratory muscle strength, daily physical activity levels, and QoL. This is due to the inability to monitor the home-based patients in real time, as this technology is expensive, demands a good internet connection at patients’ homes, and is limited and expensive in low-and middle-income countries. Furthermore, the center-based group training intensity was possibly approximately 10% above the home-based group, as the target HR was based on the CPX performed on the treadmill and the center-based group trained on the cycle ergometer.

From this pilot study, we suggest the use of technologies such as apps to monitor the number of steps and/or HR to assure training intensity and to objectively measure the patients’ adherence, particularly in non-supervised training programs. We also suggest the use of a self-reported questionnaire on patients’ motivation and satisfaction with exercise training programs to improve future personalized treatments.

## CONCLUSIONS

This home-based program can be a simple, easy-to-perform, and safe alternative to improve the functional capacity of patients with stable CHF after specialized cardiac evaluation. However, center-based training was more effective in improving inspiratory muscle strength, daily physical activity, and QoL in patients with CHF.

## AUTHOR CONTRIBUTIONS

Andrade GN, Umeda IIK, and Nakagawa NK carried out, designed, and performed the experiments. Fuchs ARCN, Mastrocola LE, Rossi-Neto JM, Moreira DAR, and Oliveira PA assisted with some measurements. André CDS performed the statistical analyses. Andrade GN and Nakagawa NK wrote the manuscript with input from all authors. Andrade GN, Umeda IIK, Mastrocola LE, Oliveira PA, Cahalin LP, and Nakagawa NK contributed to the interpretation of the results. All authors participated in reviewing the manuscript and revising its intellectual and technical content.

## Figures and Tables

**Table 1:** Demographic and clinical characteristics of home-based and center-based groups.

	Home-based (n=11)	Center-based (n=12)	*p*-value
**Age**, mean±SD (years)	59±5	61±7	0.733
**Male**, n (%)	5 (46)	9 (75)	0.147
**BMI**, mean±SD (kg/m^2^)	28±4	27±3	0.460
**LVEF**, mean±SD (%)	31±6	32±6	0.711
**NYHA II**, n (%)	10 (91)	11 (92)	0.949
**Duration of HF**, mean±SD (years)	11±8	9±9	0.557
**Etiology,** n (%)			0.278
Ischemic	2 (18)	6 (50)	0.265
Dilated	6 (55)	4 (33)	0.265
**Cardiac rhythm,** n (%)			0.339
Sinus rhythm	8 (73)	8 (67)	0.752
Atrial fibrillation	0 (0)	2 (17)	0.156
Implantable cardiac device	3 (27)	2 (17)	0.538
**Other morbidities**, n (%)			
Hypertension	10 (91)	10 (83)	0.590
Diabetes	3 (27)	3 (25)	0.901
Dyslipidemia	8 (73)	7 (58)	0.469
Myocardial infarction	4 (36)	6 (50)	0.510
**Smoking status**			0.265
Never smoker, n (%)	2 (18)	6 (50)	0.110
Ex-smoker, n (%)	8 (73)	5 (42)	0.133
Current smoker, n (%)	1 (9)	1 (8)	0.949
Mean pack-years	15±16	11±17	0.187
**Medications**, n (%)			
Amiodarone	4 (36)	4 (33)	0.879
Diuretics	10 (91)	9 (75)	0.315
Spironolactone	6 (55)	7 (58)	0.855
Beta blockers	11 (100)	12 (100)	0.999
Angiotensin-converting enzyme inhibitors	7 (64)	9 (65)	0.554
Angiotensin II receptor blockers	3 (27)	1 (8)	0.231
Digoxin	1 (9)	0 (0)	0.286
Anticoagulants	1 (9)	1 (8)	0.949
Antiplatelets	8 (73)	9 (75)	0.901
Statins	6 (55)	10 (83)	0.134

Continuous data: Mann-Whitney test; categorical data: chi-squared test BMI, body mass index; HF, heart failure; LVEF, left ventricular ejection fraction; NYHA, New York Heart Association functional classification.

**Table 2:** Functional capacity, physical activity level, and quality of life (mean values±SD) at baseline and after 12 weeks in both groups using a mixed linear model and physical activity level assessed by the IPAQ, using a non-parametric two-way analysis of variance for repeated measures.

	Home-based n=11		Centre-based n=12		*p*-value Group×Time	*p*-value Group	*p*-value Time
	Baseline	12 weeks	Baseline	12 weeks			
**Cardiopulmonary test**							
Exercise time (s)	579±144	645±119	567±148	645±90	0.872	0.842	0.059
Peak heart rate (bpm)	117±13	122±18	116±22	123±26	0.848	0.985	0.268
Peak VO_2_ (mL/kg/min)	19.2±3.9	20.0±4.2	19.5±5.3	23.2±6.1	0.085	0.448	0.011*
AT VO_2_ (mL/kg/min)	13.3±2.7	13.5±3.7	12.9±2.9	14.5±3.7	0.367	0.920	0.235
VE/VCO_2_ slope	31.6±6.4	33.4±5.8	31.4±8.0	30.7±8.1	0.196	0.594	0.657
Maximal ventilation (L/min)	51.4±15.2	62.9±28.2	58.8±30.6	74.4±33.7	0.775	0.429	0.015**
Oxygen pulse	12.1±2.7	12.1±3.1	13.6±3.9	15.2±4.0	0.108	0.129	0.130
Peak respiratory exchange ratio	1.1±0.1	1.1±0.1	1.1±0.2	1.1±0.1	0.868	0.852	0.781
**6MW distance**	460.7±63.7	500.7±86.1	513.1±77.1	538.6±72.3	0.805	0.013	0.002^£^
**Daily physical activity**							
***IPAQ***							
Sedentary, n (%)	1 (10)	0 (0)	5 (42)	0 (10)	0.299	0.428	0.001
Irregularly active, n (%)	5 (45)	1 (10)	3 (25)	0 (10)			
Active, n (%)	5 (45)	10 (90)	4 (33)	12 (100)			
***Accelerometry***							
Number of steps per day	7335±015	6873±2819	5640±2259	6541±2225	0.001^α,β^	0.258	0.832
Number of sedentary bouts per day	18.5±8.5	17.1±6.7	23.9±5.8	22.4±6.9	0.972	0.062	0.214
Sedentary bouts length (mins/bout)	19.7±10.0	18.1±7.8	26.3±7.3	23.0±7.7	0.472	0.092	0.050
Sedentary time (mins/day)	539±133	526±111	625±86	587±140	0.581	0.113	0.266
Time spent on light activities (mins/day)	360±118	333±104	299±87	323±90	0.080	0.382	0.931
Time spent on moderate-vigorous activities (mins/day)	23±16	23±9	13±9	21±16	0.199	0.218	0.190
**Health-related quality of life**							
MLHF	29±33	28±33	35±24	22±13	0.039^#^	0.965	0.023
SF-36 - Mental Health	73±32	68±33	61±23	72±16	0.001^##^	0.848	0.342

AT, anaerobic threshold; IPAQ, International Physical Activity Questionnaire; MLHF, Minnesota Living with Heart Failure Questionnaire; SF-36, Short-Form 36 questionnaire; VCO_2_, volume of exhaled carbon dioxide; VE, expiratory minute volume; VO_2_, oxygen uptake; 6 MW, 6-min walk test. *Mean difference=2.2 (95% CI: 0.5-3.9); **Mean difference=12.9 (95% CI: 2.6-23.2); ^£^ Mean difference=32.4 (95% CI: 16.5-48.4); ^α^Home-based *vs* centre-based at baseline: *p*=0.001, Mean difference=2103 (95% CI: 1229-2977); ^β^Centre-based at baseline *vs* 12 weeks: *p*=0.031, Mean difference=901 (95% CI: 67-1735); ^#^Centre-based at baseline *vs* 12 weeks: *p*=0.014, Mean difference=13.2 (95% CI: 2.3-24.1); ^##^*p*=0.031 Home-based *vs* centre-based at baseline and *p*=0.037 Centre-based at baseline *vs* 12 weeks, using Tukey multiple comparison procedure.
